# Enzyme- and gene-specific biases in reverse transcription of RNA raise concerns for evaluating gene expression

**DOI:** 10.1038/s41598-020-65005-0

**Published:** 2020-05-18

**Authors:** Nicola Minshall, Anna Git

**Affiliations:** Department of Biochemistry, Tennis Court Road, Cambridge, CB2 1QW Cambridge UK

**Keywords:** RNA, Transcriptomics

## Abstract

Reverse transcription is the first step of most analyses of gene expression, yet the quantitative biases it introduces are largely overlooked. Following a series of purpose-designed systematic experiments we cherry-pick examples of various biases introduced by reverse transcription, and alert the “gene expression community” to the pitfalls and improved practice of this fundamental technique.

## Introduction

The analysis of gene expression underpins every aspect of modern biomedical research, which in the last two decades has progressed from single-gene to genome-wide approaches. Typically, the relative abundance of specific RNAs (or more precisely, the cDNAs corresponding to those RNAs) is enumerated by quantitative PCR (real-time PCR; qPCR) or by massively parallel (next-generation; NGS) sequencing. Substantial effort has been invested in the assurance of standards for these techniques, including their linearity, positive and negative controls, normalisation of data and the recognition of inherent biochemical and computational biases.

In contrast, the initial essential step “converting” the RNA to measurable cDNA, has not been scrutinised with the same rigour, and is not subject to an accepted best practice. Although a handful of studies attempted to tackle various aspects of reverse transcription pitfalls (reviewed^1^), the prevalent practice still assumes that manufacturers’ reverse transcription (RT) protocols ensure uniform and reliable synthesis of cDNA. Over the past decade, we have accumulated anecdotal, and often contradictory, experience regarding RT non-linearity and biases.

In a series of purpose-designed experiments, we systematically demonstrate that the bias introduced by RT is far greater than is commonly assumed. Our data (Supplemental File 1) indicate that RT introduces amplicon-specific and transcriptase-specific biases which render standard calculations (*e.g*. ΔΔCq) of relative gene expression inaccurate at best and erroneous at worst. We cherry-pick examples where differential RNA integrity or saturation of the RT may falsely appear as differential expression (DE). Lastly, we propose improved practices which can easily be integrated into the MIQE guidelines^1,2^ for RT-qPCR and NGS experimental design and reporting.

## Results

**Figure 1:**
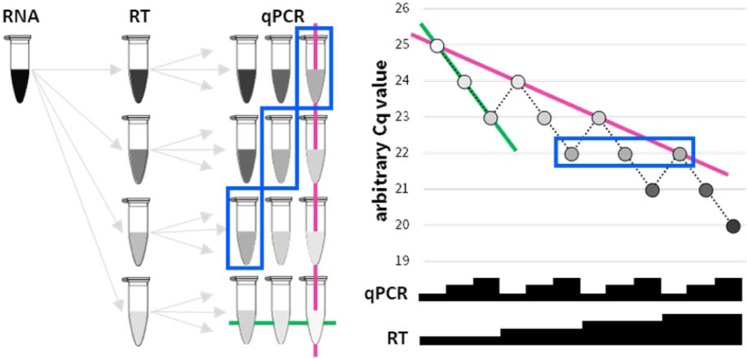
A schematic of the experimental design and Cq values of a hypothetical ideal qPCR amplification. A series of 2-fold dilutions of an RNA sample are reverse transcribed (RT), and 2-fold dilutions of each resulting cDNA are analysed by qPCR. Colour intensity of tube (left) and corresponding circle (right) represents predicted amplicon abundances. Samples boxed in blue (left and right) exemplify a set of samples expected to harbour identical Cq values. Relative input quantities are depicted as black steps (right; not to scale). Solid green lines connect samples of one ideal qPCR titration curve (-1 Cq per 2-fold increased input of cDNA in qPCR). Solid pink lines connects the samples of a similar ideal RT titration curve (-1 Cq per 2-fold increased input of RNA in RT).

In order to evaluate the effect of RT on perceived DE, we used total RNA from two cell lines, T-47D (T) and Hs578T (H). An aliquot of T RNA has been mildly chemically fragmented (Tf; Figure S1) to mimic the low-integrity (partly degraded) RNA often obtained from tissues. Four doses (2-fold: 75, 150, 300, 600 ng) of each of the three input RNAs (H, T, Tf) were reverse-transcribed using three common commercial kits. Three 2-fold dilutions of each of the resulting cDNAs were evaluated by quantitative PCR. The expectation, under ideal conditions, is that doubling the RNA input into the RT is equivalent to doubling the cDNA input into the qPCR. A hypothetical expected result is shown in Fig. 1.

We examined 8 qPCR amplicons, representing coding (a-d) and non-coding (e-h) transcripts of varying abundance, approximated by relative Cq values. They are: (a) a highly abundant eEF1A1 “exon-exon” amplicon with primers on consecutive exons spanning a 366 nt intron (b) a low abundance eEFint amplicon from this eEF1A1 intron; (c) a moderately abundant OAZ1 “exon-exon” amplicon; (d) a low abundance MVP “exon-exon” amplicon; and several non-coding RNAs (ncRNAs; (e) 18 S rRNA, (f) 5.8 S rRNA and (g) U1 snRNA), as well as (h) Alu which can be transcribed either as a stand-alone ncRNA or embedded in untranslated regions of coding genes, and which has been proposed as a measure of total RNA load instead of unreliable single-transcript RT-qPCR reference genes^3^, colloquially referred to as “housekeepers”. Notably, eEF1A1 is our best empirically-derived single reference gene for breast cancer samples, exhibiting the lowest variation across 2,000 breast tumours^4^ (observation unpublished), and later validated across multiple breast cell lines (not shown).

The key observations from our systematic analysis are general non-linearity as well as amplicon-specific and reverse transcriptase-specific biases. In general, across the entire dataset, a 2-fold increase of cDNA input into a qPCR reaction resulted in an average decrease of 0.99 in Cq value (n = 576 2-fold dilution pairs) – in line with the theoretical decrease of 1. In contrast, a 2-fold increase of RNA input into an RT reaction led to an average decrease of only 0.39 (n = 648) – a substantially lower value than the theoretical decrease. For brevity, hereafter we will refer to Cq increase per 2-fold dilution as Cq2f.

**Figure 2:**
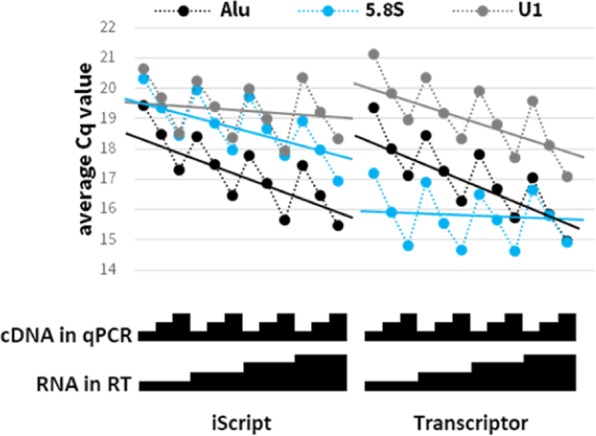
qPCR analysis of RNA H using two RT kits (iScript and Transcriptor). Experimental setup and display are as in Fig. 1. Solid lines are average RT titration slopes. For precise values, see text.

A major decision in evaluation of gene expression is the choice of reverse transcriptase enzyme or kit. This decision is typically a matter of lab tradition and cost, and is seldom evaluated when experimental designs change^5^. Figure 2 highlights kit-dependent biases in RT-qPCR analysis. It presents Cq values obtained in the analysis of Alu, 5.8 S and U1 amplicons in dilutions of RNA from a single cell line (H), reverse-transcribed using two commercial RT kits: iScript and Transcriptor. These amplicons yield similar Cq values (by proxy: similar abundance), thus ruling out major discrepancies due to stoichiometry. Predictably, the titration of all individual cDNAs into the qPCR consistently resulted in Cq changes (~1 Cq2f; compare to solid green line in Fig. 1). In contrast, the titration of RNA into iScript RT led to “compressed” but consistent Cq increase in 5.8 S (0.45 ± 0.02 Cq2f), but not U1 amplicons (0.13 ± 0.04 Cq2f), while a similar titration into Transcriptor RT led to increase in U1 (0.56 ± 0.06 Cq2f) but not 5.8 S Cq values (0.07 ± 0.12 Cq2f). The Cq values for Alu (and other tested amplicons; Supplemental File 1) behaved consistently in both RT systems (increase of 0.65 ± 0.02 and 0.73 ± 0.03 Cq2f, respectively). Similarly, among the coding mRNAs, MVP RT-qPCR is insensitive to RNA titration into the iScript RT (0.23 ± 0.09 Cq2f) while eEF1A1 RT-qPCR responds better (0.51 ± 0.09 Cq2f). Thus, applying the ΔΔCq method, there is an apparent DE (>5-fold) of MVP relative to eEF1A1 between dilutions of the same RNA. This is not due to problems with the amplicon or the target mRNAs as when using Transcriptor RT, the maximal apparent DE between RNA dilutions is 1.6-fold.

**Figure 3:**
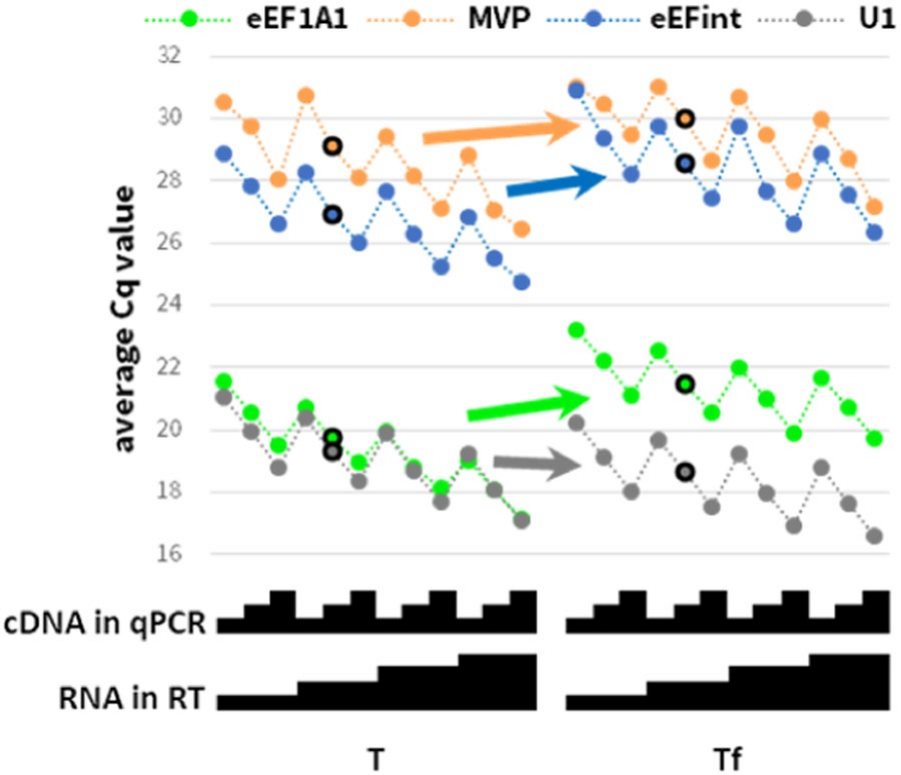
qPCR analysis of intact (T) and partly-degraded (Tf) RNA T using Transcriptor. Experimental setup and display are as in Fig. 1. Arrows exemplify the trend of change in Cq values using the highlighted dilution (outlined circles).

An added complication in studies of gene expression in patient tissues, is inconsistent RNA integrity across the sample cohort^6^. This can represent an inherent biological truth (*e.g*. native, necrotic or inflammatory RNase activity), or can be the artifact of variable sample storage and processing (*e.g*. time taken to freeze biopsies, or slower lysis of fibrotic samples). Figure 3 illustrates the biases in RT-qPCR analyses of our model RNAs of different integrity from the same sample: intact (T) and partly-degraded (Tf). As expected, most amplicons (here: MVP, eEF1A1 and eEFint) yield a slightly higher Cq value in Tf, due to the lower abundance of intact RT template (eEF1A1 Cq values increase by 2.00 ± 0.43; MVP1 by 0.94 ± 0.42; eEFint by 1.68 ± 0.28. Coloured arrows in the figure indicate the trend of change for one highlighted dilution). In contrast, the Cq values of the U1 amplicon are decreased by 0.68 ± 0.14 (grey arrow) by compromised template integrity. This can be hypothetically attributed to the structured nature of the U1 snRNA and thus higher resistance to chemical cleavage – raising possible concerns about the use of U1 and other structured RNAs as reference genes^7^. A more subtle effect is evident when normalising MVP or eEFint amplicon values to the eEF1A1 reference amplicon, yielding a false DE of ~2-fold up-regulation between intact and fragmented samples of the same RNA. Since SuperScript-IV RT-qPCR (another commercially available RT kit) analysis of the same samples was not similarly skewed, we assume that this bias is not inherent to the presented amplicons, *e.g*. 5'/3' bias in samples degraded by exonucleolytical activity *in vivo* or in preparation^6^.

**Figure 4:**
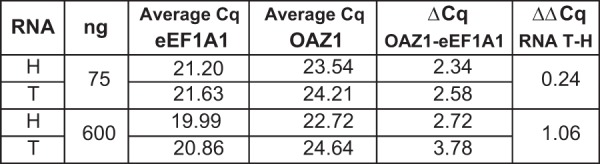
qPCR analysis of RNA from two cell lines (H and T) using iScript RT performed with either 75 or 600 ng RNA. Differential expression of OAZ1 relative to eEF1A1 is calculated using the ΔΔCq method.

We then used the data obtained in the titration experiment for a typical analysis of differential expression, as is the common practice across hundreds of research papers. Figure 4 presents the analysis of relative expression of two coding genes, OAZ1 and eEF1A1, in intact RNA from H and T cell lines. To estimate OAZ1 levels relative to the eEF1A1 reference, average Cq values obtained in quantitative PCR of iScript RT reactions performed with either 75 ng or 600 ng RNA were subjected to ΔΔCq calculation. The resulting estimate is 2-fold different between sample pairs of varying RNA input. Importantly, while the use of a single reference gene is ill-advised, and should be substituted by a rationalised or empirically-defined set of reference genes to dampen technical and biological spurious readings, the measurement of the gene of interest is always subject to biases which cannot be mitigated.

Lastly, we examined the potential contribution of the aforementioned biases on reverse transcription used in the generation of libraries for RNA-seq. To this end, we have set up a similar titration experiment using the SuperScript-II protocol and reagents from the Illumina’s TruSeq® Stranded Total RNA Sample Preparation Guide. To rule out the contribution of potential contaminants due to our RNA extraction techniques, we processed alongside H and T cell line RNAs similar quantities of Stratagene QPCR Human Reference Total RNA (U), which is a high-quality commercial control RNA recommended for quantitative PCR gene-expression analysis. Each 75–600 ng sample was tested with or without associated TruSeq fragmentation (Hf, Tf or Uf RNAs, respectively).

**Figure 5:**
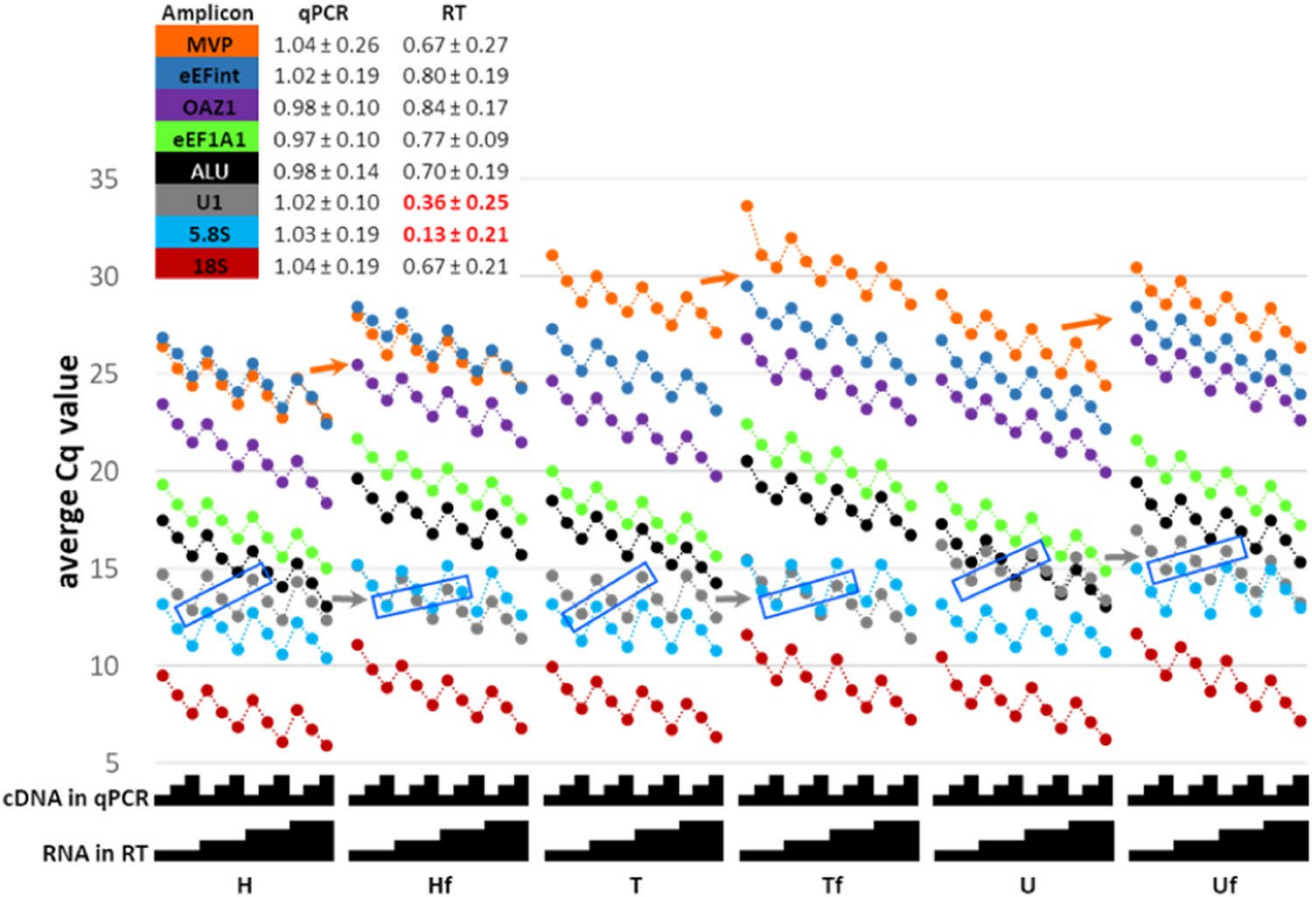
qPCR analysis of RNA from two cell lines (H and T) as well as a commercial reference (U) with or without fragmentation (fragmented: Hf, Tf and Uf). Experimental setup and display are as in Fig. 1. Amplicon colour-coding is as in Figs. 2 and 3. Samples boxed in blue exemplify sets of U1 samples expected to harbour identical Cq values (see Fig. 1). Arrows illustrate the shift in Cq of two amplicons upon fragmentation of each of the three tested RNAs.

Figure 5 summarises the average Cq values obtained in this experiment, as well as the difference in Cq obtained for 2-fold input dilutions in RT or qPCR for each amplicon. We confirm the key findings listed above. In particular, the overall average change in Cq for a 2-fold dilution of RT input into qPCR is 1.01 ± 0.17 (n = 384) while that of a 2-fold dilution of RNA input into an RT is 0.62 ± 0.31 (n = 432). The latter value can be as low as 0.13 ± 0.21 Cq2f for 5.8 S rRNA (n = 54; turquoise), despite the qPCR titration for the same amplicon showing 1.03 ± 0.19 Cq2f. The poor RT dependency of 5.8 S on RNA input is visible in all intact as well as fragmented RNAs. Interestingly, in contrast to the fragmentation used in the previous experiment (Fig. 3), the RT dependency of U1 is vastly improved upon TruSeq fragmentation (from 0.15 ± 0.16 to 0.58 ± 0.03 Cq2f; grey). Unfortunately, due to the proprietary nature of the reagents, we are unable to speculate as to the reason. Lastly, similarly to the findings of Fig. 3, amplicons are differently affected by RNA fractionation. Simplistic ΔΔCq analysis of MVP (orange) or eEFint (blue) amplicons relative to eEF1A1 (green), can yield a perceived DE of up to 2.4-fold between intact and fragmented pairs of identical RNA inputs. There is no obvious difference between the behaviour of any tested amplicon between commercial RNA (U) or those prepared in the lab, suggesting that the reported biases cannot be attributed to preparation methods.

## Discussion

These systematic data corroborate our (and colleagues’) cumulative anecdotal experience involving >100 amplicons (including several bacterial genes as well as many eukaryotic project-specific coding genes; commonly used reference genes, such as GAPDH, ACTB, HPRT1, 18 S and 5 S ribosomal RNA, U6 snRNA, U44 snoRNA; and various microRNA genes) across thousands of samples and dozens of experimental designs. Differential dose-response – and ultimately saturation – of RT reactions is far more prevalent than is commonly assumed, even within manufacturers’ usage parameters. In line with published observations of lower reproducibility of RT compared to qPCR^8^, in our experience RT biases lead to far greater errors than qPCR imperfections, especially when users adhere to MIQE-based practices for qPCR. Amplicon-dependent biases within a chosen RT system can identify non-existent DE or mask real effects. Moreover, RT is the founding step in many cutting edge technologies, such as single-cell expression analysis. Leaving its biases untested undermines such advances.

We cannot pinpoint a single factor, either in the nature of the qPCR amplicon or the RT procedure, which can alleviate all these biases. In addition to critical evaluation of available reverse transcriptases^5^, in our experience, the maximal RNA input suggested by all manufacturers is too high; and the most substantial – although by no means complete – improvement to RT biases is achieved by reducing the RNA input at the cost of higher eventual Cq values.

Many additional hypothetical factors can play a role in differential RT efficiency or imperfect dose dependence. In addition to their implication on optimal primer design^1^, secondary structures may lead to differential denaturation of RNA templates at the RT temperature. This bias can easily be minimised by avoiding one-step RT-qPCR methods^1^ and by using a thermostable RT enzyme at a higher temperature. Unfortunately, the effect of the same structures on differential degradation *in vivo* or during RNA extraction cannot be as easily moderated.

In the absence of secondary structure, primary sequence may play an under-researched role in RT efficiency. Features such as GC-content and repeat sequences are well known to cause “difficult regions” in PCR amplification, and numerous simple or proprietary additives exist to alleviate these difficulties in PCR. It stands to reason that similar effects will take place at the RT step. The use of random hexamers for priming RT throughout the entire length of RNAs poses additional challenges. These are not only dependent on local GC-content, but certain hexamer variants may be sequestered by abundant transcripts, thus causing a non-linear stoichiometry for less abundant transcripts. Manufacturers are invited to re-evaluate their protocols, perhaps using synthetic transcripts in varying mixed RNA backgrounds.

The incorporation of multiple rationalised^9^ reference “housekeeping” amplicons, as recommended by the MIQE guidelines^2^, can dampen the effect of some amplicon-specific problems. We further advocate restricting the use of ΔΔCq-like approaches based on reference amplicons to fine-tuning of minor fluctuations (<1.5-fold or ΔCq = 0.58) in RNA input. We strongly advise including reference RNA titration curves in the RT step, much like the DNA (or cDNA) titration curves required under the MIQE guidelines for correcting qPCR analyses of amplicons with differential amplification efficiency. Moreover, where sample availability and budgets allow, RT of key biological samples should not only be replicated to account for RT variability^8^, but also carried out with varying RNA inputs to ensure that perceived DE is dose-independent.

Lastly, we have used qPCR to demonstrate specific RT biases generated by kits aimed at single-gene and genome-wide readouts alike. False-positive and false-negative outcomes of biased RT may be greater on a genome-wide scale, and the RNA community will greatly benefit from an appropriately directed analysis of relevant NGS datasets. Complementary efforts from the manufacturers of RT kits are also called for.

## Materials and Methods

### RNA extraction and fragmentation

Total RNA was isolated from T-47D and Hs578T adherent breast cancer cell lines using Direct-zol (Zymo R2052), including the optional DNase treatment step. Stratagene Human Reference Total RNA (#750500), a pool of 10 human cell lines certified for use in qPCR, was purchased from Agilent. For Experiment 1, 10 µg aliquots were fragmented using RNA Fragmentation Reagents (Thermo AM8740) for 50 sec at 70 °C or 0 °C to generate matched part-fragmented or intact samples, respectively. RNA was re-purified using Direct-zol. For Experiment 2, fragmentation was carried out as part of the TruSeq Stranded Total RNA protocol (see below). Equal load and integrity of all input RNAs were confirmed by electrophoresis through an agarose gel impregnated with SYBR Safe (Thermo) followed by imaging under blue light (Figure S1).

### Reverse transcription

Aliquots of 600, 300, 150 or 75 ng of each RNA, alongside a sample containing only water (RT0; to control for contamination of reagents), were reverse-transcribed using one of four systems: Transcriptor (Roche), SuperScript IV (ThermoFisher), iScript (BioRad) or SuperScript II (ThermoFisher; using Illumina TruSeqStranded Total RNA reagents and protocols), essentially following manufacturers’ recommendations while keeping optional choices (*e.g*. primers, or pre-RT heat-denaturation of RNA) as consistent as possible. For each system, a pool of 200 ng of each of the RNAs was also treated alongside individual samples with the omission of the enzyme (RT-; to estimate possible contribution of traces of genomic DNA to quantitative PCR amplification). To minimise error (*e.g*. pipetting of small volumes), all non-template reagents were combined into master mixes.

#### Transcriptor

1 µl of 10 µM T15VN and 1.5 µl of 100 µM N6 primers were added to the RNA in a final volume of 13 µl. Following denaturation for 10 min at 65 °C, samples were snap-cooled on ice and supplemented with 4 µl 5x Transcription Reaction Buffer, 0.5 µl RiboLock RNase Inhibitor (ThermoFisher EO0381), 2 µl 10 mM dNTP and 0.5 µl Transcriptor enzyme. Reverse transcription was initiated for 10 min at 25 °C, proceeded for 30 min at 55 °C and finally terminated by incubating for 5 min at 85 °C.

#### SuperScript IV

1 µl of 10 µM T15VN, 1.5 µl of 100 µM N6 primers and 1 µl 10 mM dNTP were added to the RNA in a final volume of 13 µl. Following denaturation for 10 min at 65 °C, samples were snap-cooled on ice and supplemented with 4 µl 5x SSIV Buffer, 1 µl RiboLock RNase Inhibitor, 1 µl dithiothreitol (DTT) and 1 µl SuperScript IV enzyme. Reverse transcription was initiated for 10 min at 25 °C, proceeded for 30 min at 55 °C and finally terminated by incubating for 5 min at 85 °C.

#### iScript

RNA alone, in a final volume of 15 µl, was denatured for 10 min at 65 °C and snap-cooled on ice. Following the addition of 4 µl 5x iScript Mastermix and 1 µl iScript enzyme, reverse transcription was initiated for 10 min at 25 °C, proceeded for 30 min at 46 °C and finally terminated by incubating for 1 min at 95 °C.

#### SuperScript II

RNA alone, in a final volume of 8.5 µl, was mixed with 8.5 µl of Illumina Elute, Prime, Fragment High Mix and incubated either at 94 °C for 8 min or at 65 °C for 10 min, resulting in fragmented or intact templates, respectively. Following snap-cooling and addition of 7.2 µl of First Strand Synthesis Act D Mix and 0.8 µl SuperScript II enzyme, reverse transcription was initiated for 10 min at 25 °C, proceeded for 15 min at 42 °C and finally terminated by incubating for 15 min at 70 °C.

### Quantitative PCR

#### Experimental procedure

A 15 µl aliquot of each reverse transcription reaction was diluted 20-fold. Two additional 2-fold serial dilutions were made for all samples except RT0 and RT- samples. 8 µl quantitative PCR reactions in 384-well plates contained 4 µl diluted cDNA (equivalent to 0.2, 0.1 or 0.05 µl of the RT reaction, depending on the dilution) and 4 µl of either FAST SYBR Green Master Mix or PowerUp SYBR Green Master Mix (Applied Biosystems 4385612 or A25780, respectively; supplied as a 2x concentrate) supplemented with 1 µM each primer (listed in Table 1) to a final concentration of 500 nM primers in the reaction. Cycling was carried out on a CFX384 (Bio-Rad) or QuantStudio 5 (ThermoFisher) machine. Cycling parameters (as recommended by Applied Biosystems) included an initial denaturation at 95 °C for 20 sec, 40 cycles of 2-step amplification comprised of touch denaturation (1 sec) at 95 °C and extension with data collection at 60 °C for 20 sec, and a melt curve analysis based on slow renaturation and denaturation between 60 °C and 95 °C. Quantification cycle (Cq) was determined by the manufacturer’s software using automatic settings. Manual selection of thresholds for Cq determination introduced only insubstantial differences into the presented analyses, conclusions and recommendations.

**Table 1:** Primer sequences.

Primer	Purpose	Sequence (F)	Sequence (R)	Amplicon size cDNA (gDNA)
T15VN	Clamped oligo(dT) for reverse transcription of 3' termini of polyadenylated RNA	TTTTTTTTTTTTTTTVN V = [A, C, G] N = [A, C, G, T]	N/A	N/A
N6	Random Hexamers for reverse transcription throughout all RNA	NNNNNN N = [A, C, G, T]	N/A	N/A
eEF1A1	qPCR of eEF1A1 cDNA (exon-exon junction)	GGCATCGACAAAAGAACCAT	CCCAGGCATACTTGAAGGAG	79 (79*, 445)
eEFint	qPCR of eEF1A1 cDNA (intronic)	TGGTTGCTTCTGTAACCCAAA	CAGCCCTTAATTGGCAGTTT	79 (79)
OAZ1	qPCR of OAZ1 cDNA (exon-exon junction)	GCTCCTAAGCCTGCACAGC	GACCCGGGTTACTACAGCAG	80 (1720)
MVP/LRP	qPCR of MVP/LRP cDNA	AGGCCAAGCTAAAAGCACAG	AGCTCTCGGACCTTCTGGAC	76 (988)
18 S rRNA	qPCR of 18 S rRNA cDNA	CTGGATACCGCAGCTAGGAA	ATCATGGCCTCAGTTCCGAA	75 (75)
5.8 s rRNA	qPCR of 5.8 S rRNA cDNA	GGTGGATCACTCGGCTCGT	GCAAGTGCGTTCGAAGTGTC	102 (102)
U1	qPCR of U1 snRNA cDNA	CCATGATCACGAAGGTGGTTT	ATGCAGTCGAGTTTCCCACAT	101 (101)
Alu	qPCR of Alu cDNA	CATGGTGAAACCCCGTCTCTA	GCCTCAGCCTCCCGAGTAG	90–93 (90–93)

*Processed pseudogenes.

#### Quality control

The existence of a single end product was verified by melt-curve analysis, and the end product of selected qPCR reactions was confirmed by electrophoresis through an agarose gel impregnated with SYBR Safe (Thermo) followed by imaging under blue light (Figure S2). Alu amplification results from a large number of templates and therefore produces a broader melting peak and minor bands in addition to the main product. A sample of normal genomic DNA (Cambio CA-972-F) was analysed alongside cDNA as an additional control to ensure no cross-intron amplification. To minimise error all reactions have been set up using single- or multi-channel electronic repeat pipettors, and avoiding pipetting volumes <1 µl. No substantial readings were obtained in RT0 and RT- negative control samples.

#### Data preprocessing

All reactions were set up in triplicate. For simplicity and consistency of removing aberrant qPCR wells (*e.g*. containing dust or bubbles), downstream analyses were performed using the average of the two closest triplicates. Using a median or average of all three replicates introduced only insubstantial differences into the presented analyses, conclusions and recommendations. The standard deviation across triplicates was 0.19 ± 0.27 Cq, and across best duplicates −0.06 ± 0.10 Cq (representing duplicates with Cq difference of 0.09 ± 0.14), with high standard deviations mostly affecting readings with Cq >30 (single-molecule stochastic readings), such as negative controls. For clarity of presentation, error bars depicting the standard deviation were omitted throughout the manuscript.

### Primers

All primers were designed using the Primer3 algorithm at http://bioinfo.ut.ee/primer3-0.4.0/primer3/ using default parameters (60 °C optimal melting temperature, 18–27 nt primer) to result in a 70–110 bp amplicon suitable for fast quantitative PCR amplification.

## Supplementary information

Supplemental Figures S1-S3.

Dataset 1.

## References

[1] Bustin S, Nolan T (2017). Talking the talk, but not walking the walk: RT-qPCR as a paradigm for the lack of reproducibility in molecular research. Eur. J. Clin. Invest..

[2] Bustin SA (2009). The MIQE Guidelines: Minimum Information for publication of Quantitative real-time PCR Experiments. Clin. Chem..

[3] Marullo M (2010). Expressed Alu repeats as a novel, reliable tool for normalization of real-time quantitative RT-PCR data. Genome Biol..

[4] Curtis C (2012). The genomic and transcriptomic architecture of 2,000 breast tumours reveals novel subgroups. Nature.

[5] Stahlberg A, Kubista M, Pfaffl M (2004). Comparison of Reverse Transcriptases in gene expression analysis. Clin. Chem..

[6] Vermeulen, J. *et al*. Measurable impact of RNA quality on gene expression results from quantitative PCR. *Nucleic Acids Res*. **39**, (2011). 10.1093/nar/gkr065 PMC3089491 21317187

[7] Galiveti CR, Rozhdestvensky TS, Brosius J, Lehrach H, Konthur Z (2010). Application of housekeeping npcRNAs for quantitative expression analysis of human transcriptome by real-time PCR. RNA.

[8] Tichopad A (2009). Design and optimization of reverse-transcription quantitative PCR experiments. Clin. Chem..

[9] Eisenberg E, Levanon EY (2013). Human housekeeping genes, revisited. Trends in Genetics.

