# Supplementary material for: Enzyme- and gene-specific biases in reverse transcription of RNA raise concerns for evaluating gene expression
Source: Sci Rep. 2020 May 18;10:8151. doi: 10.1038/s41598-020-65005-0 (PMC7235240; doi:10.1038/s41598-020-65005-0)
Supplement: Supplementary file 1 — Supplemental Figures S1-S3. [file 41598_2020_65005_MOESM1_ESM.pdf]

# **Enzyme- and gene-specific biases in reverse transcription of RNA raise concerns for evaluating gene expression.**

Nicola Minshall and Anna Git

*Supplemental information (Figures S1-S3)*

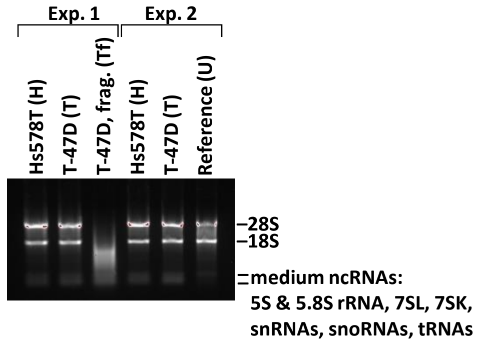


**Figure S1**: 300 ng of total RNA used in Experiments (Exp.) 1 and 2 were resolved on an agarose gel impregnated with SYBR Safe (Thermo) and visualised under blue light. The migration of 28S and 18S rRNA, and of a large number of medium-length ncRNAs is indicated. Reference: Stratagene Human Reference total RNA.

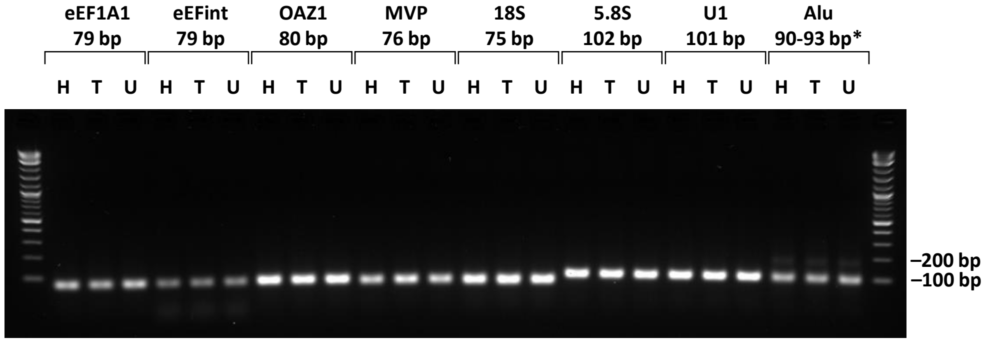


**Figure S2**: 10 µl of completed qPCR reactions (middle dilutions of RT reactions programmed with 300 ng RNA) from Experiment 2 were resolved on an agarose gel impregnated with SYBR Safe (Thermo) and visualised under blue light. The migration of 100 and 200 bp size marker is indicated. Amplicon names are listed with their predicted sizes. H – Hs578T, T – T-47D, U – Stratagene human Reference total RNA.

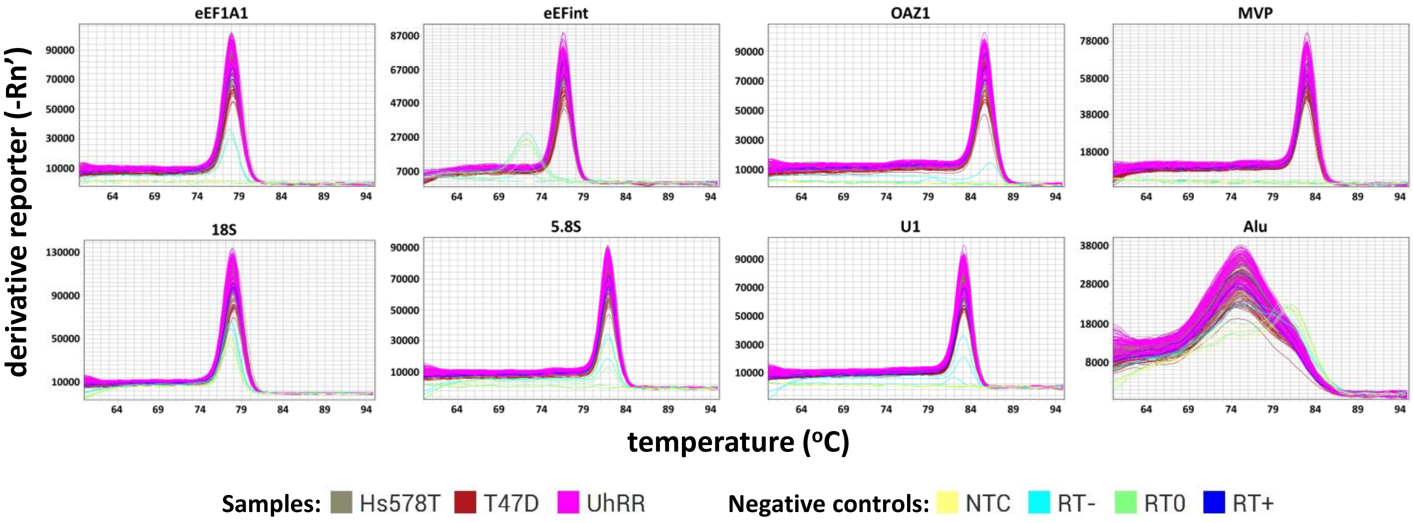


**Figure S3**: Melt curve analysis of qPCR reactions from Experiment 2.
